# Longitudinal Study of Performance on the Ruff Figural Fluency Test in Persons Aged 35 Years or Older

**DOI:** 10.1371/journal.pone.0121411

**Published:** 2015-03-23

**Authors:** Marlise E. A. van Eersel, Hanneke Joosten, Janneke Koerts, Ron T. Gansevoort, Joris P. J. Slaets, Gerbrand J. Izaks

**Affiliations:** 1 University of Groningen, University Medical Center Groningen, University Center for Geriatric Medicine, Groningen, The Netherlands; 2 University of Groningen, University Medical Center Groningen, Department of Nephrology, Groningen, The Netherlands; 3 University of Groningen, Department of Clinical and Developmental Neuropsychology, Groningen, The Netherlands; University of Granada, SPAIN

## Abstract

The Ruff Figural Fluency Test (RFFT) is a cognitive test to measure executive function. Longitudinal studies have shown that repeated testing improves performance on the RFFT. Such a practice effect may hinder the interpretation of test results in a clinical setting. Therefore, we investigated the longitudinal performance on the RFFT in persons aged 35–82 years. Performance on the RFFT was measured three times over an average follow-up period of six years in 2,515 participants of the Prevention of REnal and Vascular ENd-stage Disease (PREVEND) study in Groningen, the Netherlands: 53% men; mean age (SD), 53 (10) years. The effect of consecutive measurements on performance on the RFFT was investigated with linear multilevel regression models that also included age, gender, educational level and the interaction term consecutive measurement number x age as independent variables. It was found that the mean (SD) number of unique designs on the RFFT increased from 73 (26) at the first measurement to 79 (27) at the second measurement and to 83 (26) at the third measurement (*p*<0.001). However, the increase per consecutive measurement number was negatively associated with age and decreased with 0.23 per one-year increment of age (*p*<0.001). The increase per consecutive measurement number was not dependent on educational level. Similar results were found for the median (IQR) number of perseverative errors which showed a small but statistically significant increase with repeating testing: 7 (3–13) at the first measurement, 7 (4–14) at the second measurement and 8 (4–15) at the third measurement (*p*_trend_ = 0.002). In conclusion, the performance on the RFFT improved by repeating the test over an average follow-up period of three to six years. This practice effect was the largest in young adults and not dependent on educational level.

## Introduction

In aging adults, cognitive function changes over time. Although, the underlying mechanism is not completely understood, it is generally acknowledged that cerebrovascular and neurodegenerative changes play an important role [1,2]. Generally, one of the first changes in cognitive function occurs in the domain of executive function because executive functions are sensitive to early cognitive impairment [3,4]. Executive functions encompass a variety of higher-order cognitive processes that include planning, inhibition, cognitive flexibility, decision-making and self-monitoring, and are commonly assessed by fluency tests [3,5]. Fluency refers to the ability to generate within limited time varied verbal or non-verbal responses to a specific instruction while avoiding response repetition [6]. One test to measure non-verbal fluency is the Ruff Figural Fluency Test [6].

The Ruff Figural Fluency Test (RFFT) requires participants to draw as many different designs as possible without replicating designs [6,7]. The RFFT was designed as a variation on the first figural fluency test of Jones-Gotman and Miller [6–8], and provides information regarding different cognitive abilities such as planning strategies, divergent thinking and the ability to shift between different cognitive tasks [6,7]. The RFFT was evaluated in several populations and was found to discriminate well between healthy persons and persons with brain injury or dementia [6,9,10]. In addition, the RFFT is sensitive to changes in executive function in both young and older persons [6,11]. Yet, the RFFT may have an important limitation because for various fluency tests the performance improves substantially by repeating the test [12–14]. Clearly, such a practice effect would impair the interpretation of test results in a clinical setting. Up till now, four studies have found that performance on the RFFT might also be dependent on repeated testing [6,15–17]. However, these studies only included carefully selected and small study populations of healthy persons. More importantly, measurement of performance on the RFFT was repeated after a relatively short follow-up period of three weeks to twelve months [6,15–17]. However, in clinical practice, the follow-up period of patients with cognitive complaints is often considerably longer. It is not clear if a practice is still relevant after that period of time. Thus, it is still unknown how performances on the RFFT after repeated measurement over a longer follow-up period can be interpreted.

The aim of this study was to investigate the longitudinal performance on the RFFT by repeating the test over an average follow-up period of three and six years in a large cohort that included 2,515 community-dwelling persons aged 35 to 82 years old with different educational levels.

## Methods

### Study Population

The data of this study were collected in the third, fourth and fifth survey of the Prevention of REnal and Vascular ENd-stage Disease (PREVEND) study. The PREVEND study was initiated in 1997 to investigate prospectively the natural course of microalbuminuria and its association with renal and cardiovascular diseases in the general population [18,19]. Briefly, all habitants of the city of Groningen, the Netherlands, aged 28–75 years old, were invited to participate in the study. Finally, 8,592 participants were selected for the first survey (1997–1998) based on their urinary albumin excretion and were followed over time. The RFFT was introduced at the third survey of the PREVEND study (2003–2006). A total of 4,158 participants completed the first measurement of the RFFT. Of those, twenty-three participants (0.6%) were excluded because of incomplete demographic data [20]. Thus, the total study population included 4,135 persons, who were invited to perform the RFFT for a second time in the fourth survey (2006–2008) and for a third time in the fifth survey (2008–2012). Further details of the PREVEND study can be found in Mahmoodi *et al*. and Lambers Heerspink *et al*. [18,19].

### Ethics Statement

The PREVEND study was approved by the medical ethics committee (METc) of University Medical Center Groningen, Groningen, The Netherlands, and conducted in accordance with the guidelines of the Helsinki declaration. All participants gave written informed consent.

### Ruff Figural Fluency Test

**Fig 1:**
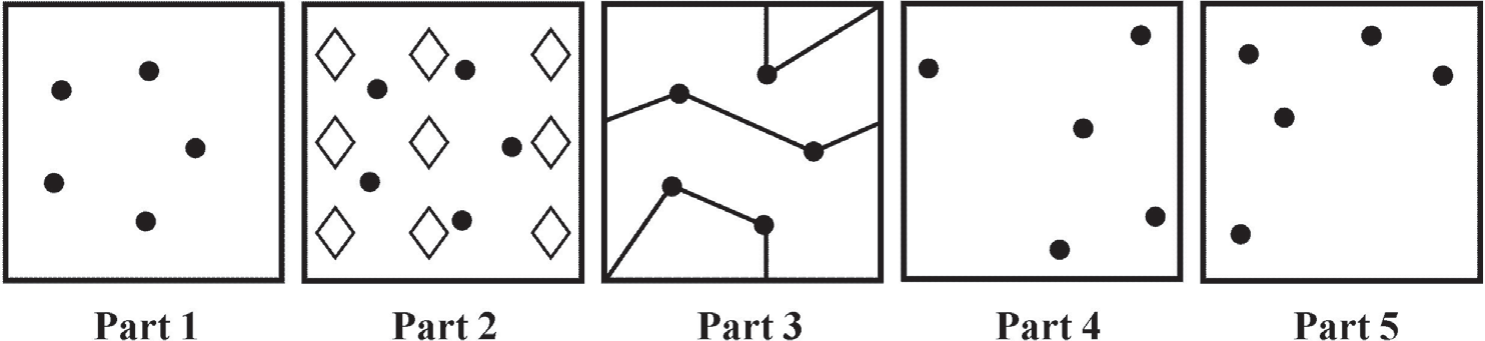
Five-dot patterns in parts 1 to 5 of the Ruff Figural Fluency Test [6,7].

The Ruff Figural Fluency Test (RFFT) is a non-verbal fluency test that measures executive function [6,7]. The test consists of five parts. Each part contains 35 five-dot patterns arranged in five columns and seven rows on a white 8.5 by 11 inches sheet of paper. Each part has a different stimulus pattern (Fig. 1). In part 1, 2 and 3, the same five-dot pattern is used but part 2 and 3 include different distractors. In part 4 and 5, the five-dot pattern is a variation of the dot pattern in part 1 and without distractors. For each part, the task is to draw as many unique designs as possible within one minute by connecting the dots while avoiding repetitions of designs. Repetitions of designs are scored as perseverative errors. Performance on the RFFT is expressed as the total number of unique designs of all five parts and the total number of perseverative errors. The relationship between the total number of unique designs and the total number of perseverative errors is sometimes expressed as error ratio: the total number of perseverative errors is divided by the total number of unique designs [6,7]. In the PREVEND study, performance on the RFFT was analyzed independently by two trained examiners. If the number of unique designs or perseverative errors as analyzed by the two examiners differed by more than two designs in one part or more than four designs in total, the analysis was repeated by a third independent examiner. Then the RFFT scores as analyzed by the two examiners who were most concordant were averaged. The Intraclass Correlation Coefficient (95% CI) between two ratings was 1.00 (0.99 to 1.00) [11].

### Other variables

Data on age at the first measurement, gender and educational level were obtained from a questionnaire. Educational level was divided into four groups according to the International Standard Classification of Education (ISCED): primary school (0 to 8 years of education; ISCED 0–1), lower secondary education (9 to 12 years of education; ISCED 2), higher secondary education (13 to 15 years of education; ISCED 3–4), and university (≥16 years of education; ISCED 5) [21].

### Statistical analysis

Parametric data are presented as mean and standard deviation (SD) and nonparametric data as median and interquartile range (IQR). Differences between unpaired observations were tested by independent-samples *t* test or, if appropriate, Mann-Whitney *U* test. Differences between paired observations were tested by paired-samples *t* test or, if appropriate, Wilcoxon signed-rank test. Differences in proportion were tested by Chi-Square test. Trends were analyzed by ANOVA for parametric data and by Kruskal-Wallis H test for nonparametric data.

The effect of repeated testing and age on performance on the RFFT was investigated by linear multilevel analysis (linear mixed model analysis). The included levels were consecutive measurement number (lowest level; value: 1, 2 or 3) and participant (highest level). The number of unique designs was the dependent variable. Consecutive measurement number, age at the first measurement (years), gender and educational level were the independent variables. Interaction between consecutive measurement number and age was investigated by entering the product term consecutive measurement number x age into the regression model. Similarly, the interaction between consecutive measurement number and educational level was investigated by entering the product term consecutive measurement number x educational level into the model. The same analyses were done with the number of perseverative errors as the dependent variable. For this variable, the analyses were repeated after log transformation because its distribution was skewed. In all regression models, the number of unique designs, the number of perseverative errors, age at the first measurement and consecutive measurement number were entered as continuous variables. Educational level and gender were entered as categorical variables. The level of statistical significance was set at 0.05. The multilevel analyses were performed using MLwiN Version 2.29 (Centre for Multilevel Modelling, University of Bristol, Bristol, UK) [22], the other analyses were performed using IBM SPSS Statistics 20.0 (IBM, Amonk, NY).

## Results

### Study population

**Fig 2:**
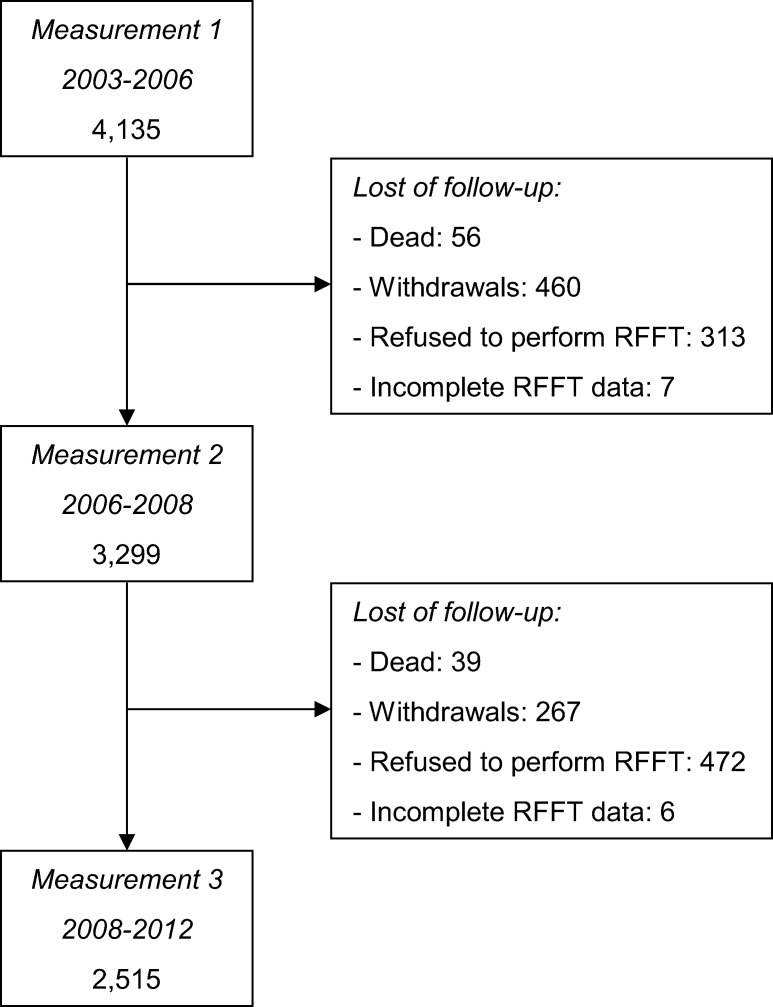
Flowchart of participants who performed the Ruff Figural Fluency Test. Mean (SD) follow-up time between measurement 1 and 2 was 2.8 (0.5) years and between measurement 2 and 3 2.7 (0.5) years. Abbreviations: RFFT, Ruff Figural Fluency Test.

A total of 2,515 participants (61%) completed the RFFT at all three measurements (Fig. 2). The mean (SD) age of all participants at the first measurement was 53 (10) years, 53% were men and 96% was of Western-European descent (Table 1). The mean (SD) follow-up time between the first and second measurement was 2.8 (0.5) years and between the second and third measurement 2.7 (0.5) years. The mean (SD) total follow-up time was 5.5 (0.7) years.

**Table 1:** Characteristics of the study population at measurement 1, 2003–2006.

	Men	Women	All
**N (%)**	1334 (53)	1181 (47)	2,515 (100)
**Age (years), mean (SD)**	53 (11)	52 (10)	53 (10)
**Age groups (years), N (%)**			
35–39	157 (12)	121 (10)	278 (11)
40–44	176 (13)	177 (15)	353 (14)
45–49	206 (15)	223 (19)	429 (17)
50–54	236 (18)	218 (18)	454 (18)
55–59	189 (14)	195 (17)	384 (15)
60–64	131 (10)	105 (9)	236 (10)
65–69	107 (8)	73 (6)	180 (7)
70–74	92 (7)	53 (5)	145 (6)
≥75	40 (3)	16 (1)	56 (2)
**Educational level, N (%)**			
Primary school	89 (7)	77 (7)	166 (7)
Lower secondary education	321 (24)	363 (31)	684 (27)
Higher secondary education	403 (30)	298 (25)	701 (28)
University	521 (39)	443 (37)	964 (38)
**Performance on the RFFT**			
Number of unique designs, mean (SD)	73 (26)	73 (25)	73 (26)
Number of perseverative errors, median (IQR)	7 (3–12)	7 (4–15)	7 (3–13)

Abbreviations: RFFT, Ruff Figural Fluency Test; SD, standard deviation; IQR, interquartile range.

Participants who did not perform the RFFT at the second or third measurement were older (mean [SD] age, 58 [13] vs. 53 [10] years; *p*<0.001) and had a lower educational level (*p*<0.001). There was no difference in gender (*p* = 0.16). The main reasons for nonperformance were refusal to participate (19%), withdrawal from the PREVEND study (18%) and death (2%).

### Unique designs

At the first measurement, the mean (SD) number of unique designs was 73 (26) in the total study population. The number of unique designs was negatively associated with age (*p*_trend_<0.001)(Table 2), and positively associated with educational level (*p*_trend_<0.001)(Table 3). Similar results were found at the second and third measurement.

**Table 2:** Longitudinal performance on the Ruff Figural Fluency Test per age group.

Performance on the RFFT	Consecutive measurement (period)			*p* value for trend^a^
	1 (2003–2006)	2 (2006–2008)	3 (2008–2012)	
**Number of unique designs, mean (SD)**				
35–39 years	91 (23)^a^ ^,^ ^b^	102 (23)^a^ ^,^ ^b^	107 (24)^a^ ^,^ ^b^	<0.001
40–44 years	83 (25)	92 (24)	98 (25)	<0.001
45–49 years	80 (24)	86 (24)	93 (24)	<0.001
50–54 years	75 (24)	79 (23)	84 (24)	<0.001
55–59 years	68 (23)	74 (23)	77 (24)	<0.001
60–64 years	63 (22)	66 (20)	67 (22)	0.08
65–69 years	55 (19)	57 (21)	57 (19)	0.48
70–74 years	50 (18)	52 (18)	52 (18)	0.56
≥75 years	50 (17)	53 (19)	49 (21)	0.47
All	73 (26)	79 (27)	83 (28)	<0.001
**Number of perseverative errors, median (IQR)**				
35–39 years	6 (3–11)^a^ ^,^ ^c^	8 (4–12)^a^ ^,^ ^c^	9 (4–15)^a^ ^,^ ^b^	0.006
40–44 years	7 (4–14)	9 (5–14)	10 (5–17)	0.006
45–49 years	7 (4–12)	8 (4–16)	8 (4–16)	0.09
50–54 years	7 (4–15)	7 (4–15)	9 (4–16)	0.11
55–59 years	7 (3–15)	7 (3–14)	7 (4–14)	0.77
60–64 years	7 (3–13)	8 (4–14)	9 (3–17)	0.38
65–69 years	7 (3–15)	7 (3–16)	7 (4–14)	0.60
70–74 years	6 (3–11)	7 (3–16)	5 (3–11)	0.38
≥75 years	8 (5–15)	6 (3–10)	7 (2–10)	0.10
All	7 (3–13)	7 (4–14)	8 (4–15)	0.002

Abbreviations: RFFT, Ruff Figural Fluency Test; SD, standard deviation; IQR, interquartile range.

^a^ Trends across consecutive measurements and age groups were analyzed by ANOVA for the number of unique designs and by Kruskal-Wallis H test for the number of perseverative errors.

^b^ *p* value for trend across age groups is <0.001.

^c^ Trend across age groups is not statistically significant.

**Table 3:** Longitudinal performance on the Ruff Figural Fluency Test per educational level.

Performance on the RFFT	Consecutive measurement (period)			*p* value for trend^a^
	1 (2003–2006)	2 (2006–2008)	3 (2008–2012)	
**Number of unique designs, mean (SD)**				
Primary school	51 (20)^a^ ^,^ ^b^	56 (23)^a^ ^,^ ^b^	56 (24)^a^ ^,^ ^b^	0.06
Lower secondary education	61 (22)	66 (23)	70 (25)	<0.001
Higher secondary education	73 (24)	79 (24)	83 (26)	<0.001
University	85 (23)	92 (24)	96 (25)	<0.001
**Number of perseverative errors, median (IQR)**				
Primary school	7 (3–15)^a^ ^,^ ^c^	7 (3–15)^a^ ^,^ ^d^	7 (3–15)^a^ ^,^ ^c^	0.79
Lower secondary education	7 (3–15)	8 (4–16)	8 (4–17)	0.43
Higher secondary education	7 (4–14)	7 (4–14)	8 (4–15)	0.35
University	7 (4–12)	7 (4–14)	8 (4–15)	0.001

Abbreviations: RFFT, Ruff Figural Fluency Test; SD, standard deviation; IQR, interquartile range.

^a^ Trends across consecutive measurements and educational levels were analyzed by ANOVA for the number of unique designs and by Kruskal-Wallis H test for the number of perseverative errors.

^b^ *p* value for trend across educational levels is <0.001.

^c^ Trend across educational levels is not statistically significant.

^d^ *p* value for trend across educational levels is <0.01.

During follow-up, the number of unique designs increased and was dependent on consecutive measurement number (Table 2). In the total study population, the mean (SD) number of unique designs increased to 79 (27) at the second measurement and to 83 (26) at the third measurement (*p*_trend_<0.001).

**Fig 3:**
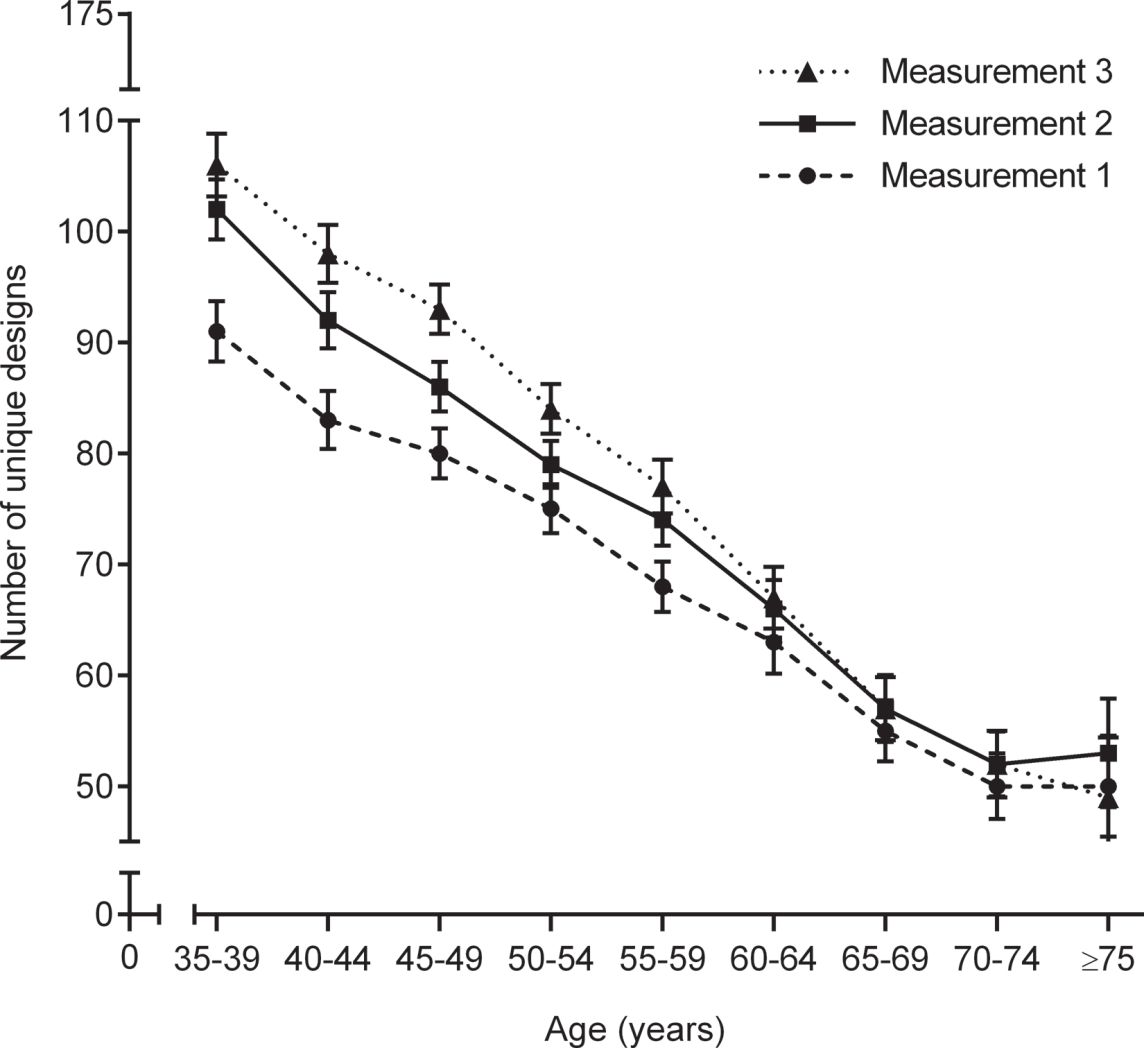
Mean number of unique designs per measurement dependent on age at baseline (measurement 1). Bars represent 95% confidence intervals.

The increase in the number of unique designs during follow-up was also dependent on age (Fig. 3). The mean difference in the number of unique designs between the first and third measurement diminished from +16 (95%CI, +14 to +18; *p*<0.001) in persons aged 35–39 years to -1 (95%CI, -6 to +4; *p* = 0.59) in persons aged 75 years or older (Table 2). This was confirmed by multilevel analysis that did not only show a statistically significant effect for consecutive measurement number and age, but also for the interaction between consecutive measurement number and age. The increase in number of unique designs between two measurements decreased with 0.23 per one-year increment of age (*p*<0.001)(Table 4).

**Table 4:** Linear multilevel regression analysis of performance on the Ruff Figural Fluency Test: unique designs.

	Model 1^b^			Model 2^c^			Model 3^d^		
	B	95%CI	*p*	B	95%CI	*p*	B	95%CI	*p*
**Age (years)**	−1.13	−1.20 to −1.05	<0.001	−1.13	−1.20 to −1.05	<0.001	−0.66	−0.76 to −0.56	<0.001
**Gender**									
Men	*Ref.*	*Ref.*		*Ref.*	*Ref.*		*Ref.*	*Ref.*	
Women	1.39	−0.03 to 2.80	0.06	1.39	−0.03 to 2.80	0.06	1.39	−0.03 to 2.80	0.06
**Educational level**									
Primary school	*Ref.*	*Ref.*		*Ref.*	*Ref.*		*Ref.*	*Ref.*	
Lower secondary education	6.60	3.53 to 9.67	<0.001	6.60	3.53 to 9.67	<0.001	6.60	3.53 to 9.67	<0.001
Higher secondary education	13.01	9.88 to 16.13	<0.001	13.01	9.88 to 16.13	<0.001	13.01	9.88 to 16.13	<0.001
University	25.31	22.26 to 28.37	<0.001	25.31	22.26 to 28.37	<0.001	25.31	22.26 to 28.37	<0.001
**Measurement^a^**	-	-	-	4.88	4.50 to 5.27	<0.001	17.11	15.15 to 19.07	<0.001
**Measurement^a^ x age**	-	-	-	-	-	-	−0.23	−0.27 to −0.20	<0.001

Abbreviations: B, unstandardized B-coefficient; CI, confidence interval; Ref, reference category.

^a^ Consecutive measurement number.

^b^ For model 1: −2*log likelihood 65821.57.

^c^ For model 2: −2*log likelihood 65241.34.

^d^ For model 3: −2*log likelihood 65088.13.

The increase in the number of unique designs during follow-up was not dependent on educational level. In the raw data, the mean difference in the number of unique designs between first and third measurement gradually increased from +5 (95%CI, +2 to +8; *p* = 0.001) in persons educated at primary school level to +11 (95%CI, +10 to +12; *p*<0.001) in persons educated at university level (Table 3). However, after adjustment for age in multilevel analysis, there was no statistically significant interaction between consecutive measurement number and educational level (data not shown).

### Perseverative errors

At the first measurement, the median (IQR) number of perseverative errors was 7 (3–13) in the total study population. The number of perseverative errors was not dependent on age (*p*_trend_ = 0.11)(Table 2), or educational level (*p*_trend_ = 0.18)(Table 3).

During follow-up, the number of perseverative errors increased and was dependent on consecutive measurement number (Table 2). In the total study population, the median (IQR) number of perseverative errors increased to 7 (4–14) at the second measurement and to 8 (4–15) at the third measurement (*p*_trend_ = 0.002). The median (IQR) error ratio did not change over time: 0.10 (0.05–0.19) at the first measurement, 0.10 (0.05–0.18) at the second measurement and 0.10 (0.05–0.19) at the third measurement (*p* = 0.53).

**Fig 4:**
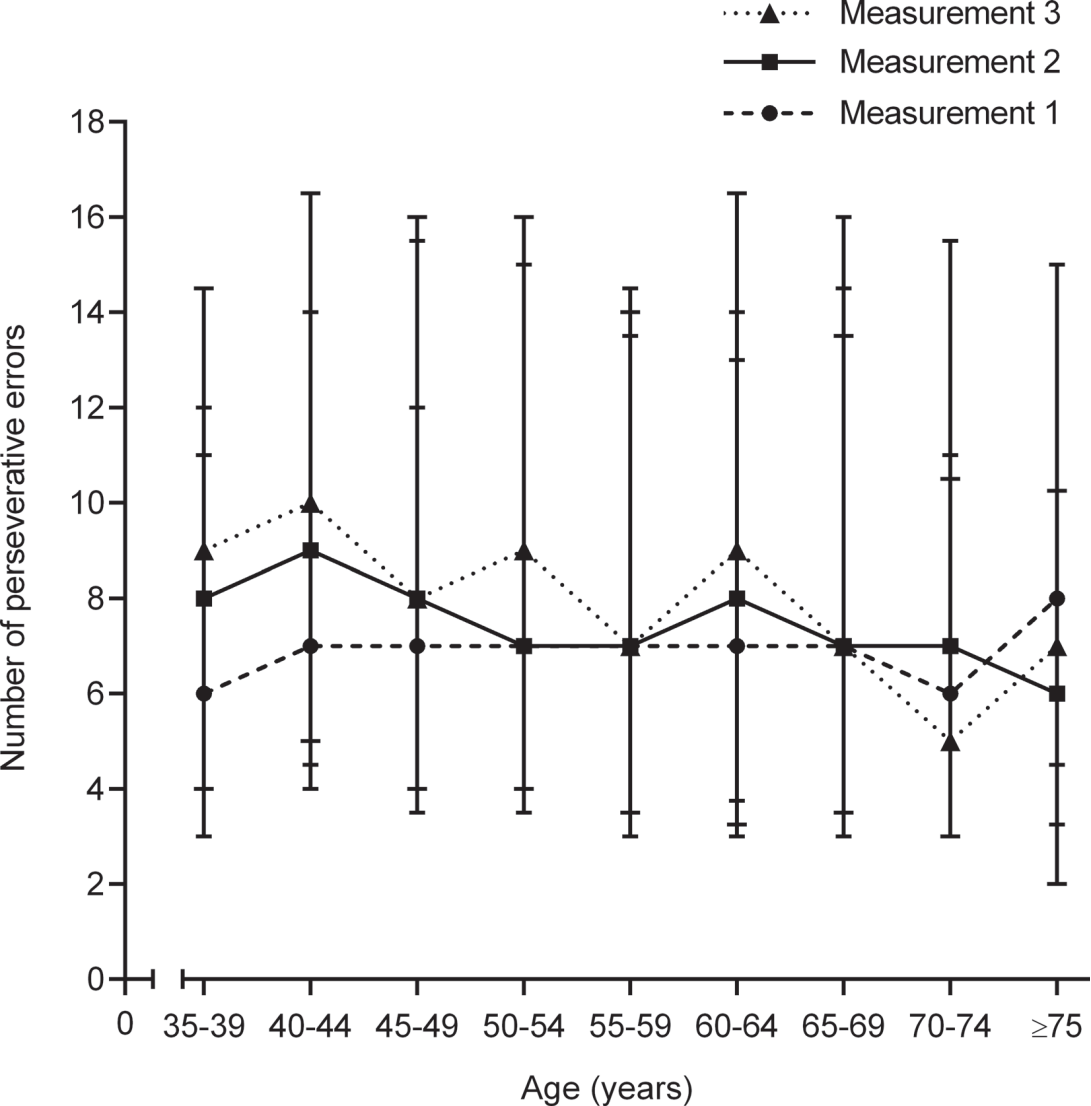
Median number of perseverative errors per measurement dependent on age at baseline (measurement 1). Upper bars represent 75th percentile, lower bars represent 25th percentile.

The increase in the number of perseverative errors during follow-up was also dependent on age (Fig. 4). The mean difference in the number of perseverative errors between the first and third measurement diminished from +2 (95%CI, +0 to +4; *p* = 0.02) in persons aged 35–39 years to -4 (95%CI, -9 to +1; *p* = 0.12) in persons aged 75 years or older. This was confirmed by multilevel analysis that did not only show a statistically significant effect for consecutive measurement number and age, but also for the interaction between consecutive measurement number and age. The increase in number of perseverative errors between two measurements decreased with 0.05 per one-year increment of age (*p* = 0.002)(Table 5).

**Table 5:** Linear multilevel regression analysis of performance on the Ruff Figural Fluency Test: perseverative errors.

	Model 1^b^			Model 2^c^			Model 3^d^		
	B	95%CI	*p*	B	95%CI	*p*	B	95%CI	*p*
**Age (years)**	−0.02	−0.06 to −0.03	0.51	−0.02	−0.06 to −0.03	0.51	0.08	0.00 to 0.15	0.04
**Gender**									
Men	*Ref.*	*Ref.*		*Ref.*	*Ref.*		*Ref.*	*Ref.*	
Women	2.64	1.76 to 3.52	<0.001	2.64	1.76 to 3.52	<0.001	2.64	1.76 to 3.52	<0.001
**Educational level**									
Primary school	*Ref.*	*Ref.*		*Ref.*	*Ref.*		*Ref.*	*Ref.*	
Lower secondary education	1.35	−0.55 to 3.26	0.16	1.35	−0.55 to 3.26	0.16	1.35	−0.55 to 3.26	0.16
Higher secondary education	−0.25	−2.19 to 1.69	0.80	−0.25	−2.19 to 1.69	0.80	−0.25	−2.19 to 1.69	0.80
University	−1.24	−3.14 to 0.66	0.20	−1.24	−3.14 to 0.66	0.20	−1.24	−3.14 to 0.66	0.20
**Measurement^a^**	-	-	-	0.10	−0.22 to 0.41	0.55	2.54	0.92 to 4.16	0.002
**Measurement^a^ x age**	-	-	-	-	-	-	−0.05	−0.08 to −0.02	0.002

Abbreviations: B, unstandardized B-coefficient; CI, confidence interval; Ref, reference category.

^a^ Consecutive measurement number.

^b^ For model 1: −2*log likelihood 60760.87.

^c^ For model 2: −2*log likelihood 60760.52.

^d^ For model 3: −2*log likelihood 60751.38.

The increase in the number of perseverative errors during follow-up was not dependent on educational level. In the raw data, the mean difference in the number of perseverative errors between the first and third measurement gradually increased from-1 (95%CI, -4 to +2; *p* = 0.58) in persons educated at primary school level to +1 (95%CI, +0 to +2; *p* = 0.01) in persons educated at university level. However, after adjustment for age in multilevel analysis, there was no statistically significant interaction between consecutive measurement number and educational level (data not shown). Essentially similar results were found when the number of perseverative errors was log transformed and the analysis was repeated (data not shown).

## Discussion

In this large community-based cohort, the performance on the RFFT improved significantly by repeating the test over an average follow-up period of three to six years. Not only the number of unique designs increased but also the number of perseverative errors increased. Interestingly, the change in number of unique designs and perseverative errors between two measurements decreased with increasing age and was not dependent on educational level.

The results in this study were comparable to the findings in two other studies by Ruff *et al*. and Basso *et al*. [6,15]. In all studies, the number of unique designs increased by repeating the RFFT. Surprisingly, the increase in number of unique designs was similar in the three study populations although the duration of follow-up was clearly different [6,15]. The duration of follow-up in the study of Ruff *et al*. was six months and in the study of Basso *et al*. twelve months while the duration of follow-up was in our study three to six years. Therefore, it can be assumed that repeating the RFFT causes a practice effect that is independent of the duration of follow-up between two consecutive measurements. In theory, it is also possible that the increase of unique designs by repeating the RFFT was (partly) caused by a practice effect in analyzing the performance on the RFFT by the examiners. However, in our study, the group of examiners was different for each consecutive measurement. Therefore, it is highly likely that repeating the RFFT causes a practice effect in performing the test. Interestingly, this practice effect persisted three to six years after the first measurement of the RFFT. On the other hand, this longstanding practice effect was not only found for the RFFT but also for other cognitive tests assessing the domains of memory and executive functions, such as the Verbal Learning Test (VLT) and the Stroop Color-Word Test (SCWT) [23–25]. Salthouse *et al*. even found that practice effects were detectable up to seven years after the first measurement of cognitive function [25]. Thus, our study is the first to show that the practice effect in performing the RFFT can persist at least three to six years after the first measurement.

Practice effects are a well-known finding when repeating neuropsychological tests [14]. It is generally assumed that practice effects can be ascribed to different factors such as reduced anxiety for or familiarity with the test, memory of specific test items or previous responses, and learning or improving test strategies [12,14,26]. Practice effects appear in several cognitive tests which assess various cognitive domains like memory, attention and executive functioning [12–14]. Several factors might especially contribute to the practice effect in the RFFT. First, the instruction to the RFFT is rather long and comprises several essential elements. Therefore, the task may seem complicated when hearing the instruction for the first time. It is likely that the instruction becomes better understood while performing the test and that better understanding of the instruction leads to better performance. Second, persons who performed the RFFT for a second time probably remember several designs from the first time. It is plausible to assume that the memory of designs drawn at the first measurement also contributed to the increase in performance on the RFFT at the subsequent measurements. Third, persons may discover strategies to improve their performance while executing the RFFT. In general, there are two different strategies to perform the RFFT: rotation and enumeration [7]. In rotation, the basic design is unchanged (for example, one line connects two dots) but its orientation is systematically rotated in each subsequent five-dot pattern of the test. In enumeration, the orientation of the basic design is unchanged but the design is systematically extended by connecting a new line to the previous one in each subsequent five-dot pattern of the test [7]. Learning and improving these strategies at repeated measurements of the RFFT probably leads to an increase in the number of unique designs per part and overall performance on the RFFT [16,27].

Practice effects have not been found for all figural fluency tests. When the Design Fluency Test (DFT) which also requires the production of unique designs under time constraints [8], was repeated after more than five years, performance had decreased across time [28]. Although this might be due to different test characteristics of the RFFT and DFT, there are several other explanations. The follow-up period in the study of the DFT, for example, was almost twice the follow-up period between the first and second measurement in our study [28]. It is likely that practice effects that occurred, at least partially, decreased during this period. Furthermore, it is likely that the participants in the study of the DFT underwent structural and functional brain changes during the long follow-up period which might have resulted in cognitive decline counterbalancing possible practice effects. We think that currently a practice effect of the DFT cannot be excluded.

Interestingly, the practice effect and the increase in performance on the RFFT at the consecutive measurements decreased with increasing age. This was in contrast with the studies of Ruff *et al*. and Basso *et al*., which did not show that the practice effect was dependent on age. A possible explanation for these divergent findings is the relatively smaller and more selected study populations in the studies of Ruff *et al*. and Basso *et al*. [6,15]. These studies also included a relatively small number of elderly people. In our study, the practice effect clearly decreased in persons aged 65 years or older but the study of Basso *et al*. included only participants aged 20–59 years old while the study of Ruff *et al*. had only 27 participants in the age group 55–70 years [6,15]. This negative association between practice effect and age was not only found for the RFFT but also for other cognitive tests that are commonly used to assess executive functions such as the Stroop Color-Word Test, Trail-Making Test part B and Wisconsin Card Sorting Test [14,23,29]. For most tests, the negative association between practice effect and age was only analyzed for two consecutive measurements [14,23,29]. However, in this study, we found that the practice effect increased further between the second and third measurement. It has been suggested that such longitudinal changes in the performance on cognitive tests are not dependent on the interval between measurements but are largely attributable to learn new strategies or to reduce anxiety when performing a test [30]. It is likely that these abilities decrease with increasing age due to the accumulation of age-related cerebral changes such as neurodegenerative and vascular lesions [31].

Notably, the number of perseverative errors also increased by repeating the RFFT. Although this increase was small, it was proportional to the increase in the number of unique designs because the error ratio did not change over time. This increase of perseverative errors was unexpected as it was assumed that repeating the RFFT would cause a practice effect and improve performance not only by the production of more unique designs but also by avoidance of perseverative errors at the consecutive measurements. However, similar results were found by Ruff *et al*. [6]. This was in contrast with another figural fluency test, the Five-Point Test (FPT), in which the number of perseverative errors decreased by repeating the test [32–34]. A possible explanation for the difference between these figural fluency tests in the change of perseverative errors is that the FPT is a simpler test to perform [34,35]. The FPT consists of only one part containing 40 five-dot patterns without distractors while the RFFT has five parts each one containing 35 five-dot patterns and different stimulus patterns [6,7,35]. As a result, the RFFT is more difficult test in respect to fluid thinking, shifting cognitive sets and planning strategies, which unsurprisingly leads to more perseverative errors.

These findings have important implications for clinical care as it is common practice to repeat a cognitive test to monitor recovery or progress of cognitive impairment. Our data show that in young persons even apparently large improvements in performance on the RFFT at repeated testing may be the result of a practice effect and not the result of recovery. Similarly, a stable performance on the RFFT at repeated testing does most likely not reflect a stable course of disease but progress. Although this problem is also present in older persons, it is much smaller in this age group. Thus, changes in performance on the RFFT at repeated testing should be interpreted differently for young and old patients.

Some limitations of this study have to be noted. First, the drop-out of participants was relatively high in our study and participants who did not perform the RFFT at the second and third measurement were older and had a lower educational level. This selection bias could have caused some overestimation of the practice effect in the older age groups. However, our study was the first to show a practice effect in the elderly of the general population as the four other longitudinal studies on repeated testing of the RFFT only included a relatively small number of strongly selected older persons aged 70 years or younger [6,15–17]. Second, in contrast to the other longitudinal studies [6,15–17], persons with neurological and psychiatric disorders were not excluded from our study. Therefore, it is likely that the health status of our study population was worse which might have led to an underestimation of the practice effect. On the other hand, our study was a better reflection of the health status of the general population and our findings were comparable to the findings of the other studies. It is generally recommended that the repeated performance of an older patient with cognitive complaints is compared to the repeated performance of older persons from the general population who are not selected on health criteria. In older persons exclusion by health criteria mostly leads to small and selected reference samples [36].

The present study also has several strengths. Our study was based on a large community-based cohort with a wide age-range and included a large number of both young and elderly people. Most importantly, our study had a long follow-up period of three to six years, which reflects the follow-up period of patients with cognitive complaints in clinical practice.

In conclusion, in this study, the performance on the RFFT improved if the test was repeated over an average follow-up period of three to six years. This practice effect was dependent on age and decreased with increasing age. The practice effect was not dependent on educational level.
